# A Data-Driven Measure of Effective Connectivity Based on Renyi's α-Entropy

**DOI:** 10.3389/fnins.2019.01277

**Published:** 2019-11-26

**Authors:** Ivan De La Pava Panche, Andres M. Alvarez-Meza, Alvaro Orozco-Gutierrez

**Affiliations:** ^1^Automatic Research Group, Faculty of Engineering, Universidad Tecnológica de Pereira, Pereira, Colombia; ^2^Signal Processing and Recognition Group, Department of Electrical and Electronic Engineering, Universidad Nacional de Colombia, Manizales, Colombia

**Keywords:** transfer entropy, kernel methods, Renyi's entropy, connectivity analysis, data-driven approach

## Abstract

Transfer entropy (TE) is a model-free effective connectivity measure based on information theory. It has been increasingly used in neuroscience because of its ability to detect unknown non-linear interactions, which makes it well suited for exploratory brain effective connectivity analyses. Like all information theoretic quantities, TE is defined regarding the probability distributions of the system under study, which in practice are unknown and must be estimated from data. Commonly used methods for TE estimation rely on a local approximation of the probability distributions from nearest neighbor distances, or on symbolization schemes that then allow the probabilities to be estimated from the symbols' relative frequencies. However, probability estimation is a challenging problem, and avoiding this intermediate step in TE computation is desirable. In this work, we propose a novel TE estimator using functionals defined on positive definite and infinitely divisible kernels matrices that approximate Renyi's entropy measures of order α. Our data-driven approach estimates TE directly from data, sidestepping the need for probability distribution estimation. Also, the proposed estimator encompasses the well-known definition of TE as a sum of Shannon entropies in the limiting case when α → 1. We tested our proposal on a simulation framework consisting of two linear models, based on autoregressive approaches and a linear coupling function, respectively, and on the public electroencephalogram (EEG) database BCI Competition IV, obtained under a motor imagery paradigm. For the synthetic data, the proposed kernel-based TE estimation method satisfactorily identifies the causal interactions present in the data. Also, it displays robustness to varying noise levels and data sizes, and to the presence of multiple interaction delays in the same connected network. Obtained results for the motor imagery task show that our approach codes discriminant spatiotemporal patterns for the left and right-hand motor imagination tasks, with classification performances that compare favorably to the state-of-the-art.

## 1. Introduction

The functional interaction of neural assemblies distributed across different brain regions underlies many cognitive and perceptual processes (Bastos and Schoffelen, 2016). Therefore, understanding such processes, and brain function at large, requires identifying the flow of information within networks of connected neural assemblies, instead of solely focusing on the activity of specific brain regions in isolation (Sakkalis, 2011; Weber et al., 2017). The analysis of the interactions mentioned above is carried out through brain connectivity measures (Friston, 2011). These measures can be subdivided into two categories based on whether they quantify the direction of the neural interactions (Sakkalis, 2011; Bastos and Schoffelen, 2016). On the one hand, non-directed functional connectivity aims to capture statistically significant interdependencies among the signals registering the activity of different neural assemblies, without determining their direction. On the other hand, directed connectivity, commonly referred to as effective connectivity, measures the influence that a neural assembly has over another one, establishing statistical causation from their signals, and hence a direction for their interaction. Effective connectivity is of particular importance in neuroscience because a large part of the brain activity is endogenous and establishing physical causality among the neural systems supporting that activity is extremely difficult (Vicente et al., 2011). So statistical causality, based on the premise that a cause precedes its effect, becomes a valuable tool to decipher multiple aspects of brain function (Seth et al., 2015; Bastos and Schoffelen, 2016).

In general, effective connectivity is assessed through measures that are either based on a model of the process generating the data, or on approaches based on information theory (Vicente et al., 2011). The former includes methods such as Granger causality (GC) and its variants, and dynamic causal modeling (DCM) (Friston, 2011; Seth et al., 2015); while the latter relies on the concept of information transfer or transfer entropy (TE) (Schreiber, 2000). While GC and DCM are widely used in neuroscience, TE has gained increasing attention in the literature (Timme and Lapish, 2018), because of the advantages it offers as compared with other effective connectivity measures. Unlike classic GC, TE can capture high order correlations, and it is well suited to detect purely nonlinear interactions in the data, which are believed to be part of brain activity on many spatial and temporal scales (Weber et al., 2017). Although DCM can capture nonlinear interactions too, it requires some *a priori* knowledge on the input of the system and on the target connectivity network, which is not always available (Vicente et al., 2011); in this sense, TE is model free. As an information theoretic quantity, TE does not need an initial hypothesize about the interactions present in the data (Timme and Lapish, 2018), so it is a particularly useful tool for exploratory analysis. However, like all other information theoretic quantities, TE is defined in terms of the probability distributions of the system under study, that in practice need to be estimated from data. Probability estimation is a challenging task, and it can significantly affect the outcome of information theory analyses, including the computation of TE (Giraldo et al., 2015; Cekic et al., 2018; Timme and Lapish, 2018). Current methods that successfully estimate TE are based on a local approximation of the probability distributions from nearest neighbor distances (Kraskov et al., 2004; Lindner et al., 2011), or on symbolization schemes that then allow the probabilities to be estimated from the symbols' relative frequencies (Dimitriadis et al., 2016). Nonetheless, obtaining TE directly from data, without the intermediate step of probability estimation, as has been achieved for other information theoretic quantities (Giraldo et al., 2015), is desirable.

In this work, we propose a data-driven TE estimator that sidesteps the need to obtain the probability distribution underlying the data. We begin by expressing TE as a linear combination of Renyi's entropy measures of order α (Rényi, 1961; Principe, 2010), instead of using the standard definition in terms of Shannon entropies. Renyi's entropy is a mathematical generalization of the concept of Shannon entropy. It corresponds to a family of entropies that, because of its functional dependence on the parameter α, can emphasize either mean behavior and slowly change features in the data, or rare, uncommon events (Gao et al., 2011; Giraldo et al., 2015). This flexibility gives Renyi's entropy an advantage when it comes to analyzing data from biomedical systems (Liang et al., 2015), and has been exploited in neuroscience studies, for instance, to better characterize the randomness of EEG signals in childhood absence epilepsy (Mammone et al., 2015), and to track EEG changes associated with different anesthesia states (Liang et al., 2015). Renyi's entropy has also been employed as an EEG feature extraction strategy in automatic systems for the diagnosis of epilepsy (Acharya et al., 2015), and for the assessment of cognitive workload (Zarjam et al., 2013). Afterward, we approximate Renyi's entropy through a functional defined on positive definite and infinitely divisible kernels matrices, introduced in Giraldo et al. (2015). The obtained estimator computes TE directly from the kernel matrices that, in turn, capture the similarity relations among data. Also, because of the definition of Renyi's entropy, the proposed approach encompasses the conventional formulation of TE as a sum of Shannon entropies in the limiting case when α → 1.

In order to test our proposal, we use a simulation framework consisting of two linear models, based on autoregressive approaches and a linear coupling function, respectively, and on a real-world task from the public EEG database BCI Competition IV, obtained under motor imagery (MI) paradigm. In particular, we aimed to test whether our method fulfills the requirements established in Vicente et al. (2011) for a TE estimator suited for neuroscience data. Namely, it must be robust to moderate levels of noise, it must rely on a limited number of data samples, and it must be reliable when dealing with high dimensional spaces. For the synthetic data, the proposed kernel-based TE estimation method successfully detects the presence and direction of the causal interactions defined in the models. Additionally, it displays robustness to varying noise levels and data sizes, in terms of the available data samples, and to the presence of multiple interaction delays in the same connected network. Finally, the results for the MI data show that our approach codes discriminant spatiotemporal patterns for the left and right-hand motor imagination tasks, that are in accordance with the temporal structure of the MI paradigm.

The remainder of the paper is organized as follows: section 2 reviews the theoretical foundations of TE, section 3 presents the concept of information theoretic learning and introduces our approach to TE estimation, section 4 describes the three experiments carried out to evaluate the performance of our method, section 5 shows our results and their accompanying discussion, and finally, section 6 contains our conclusions.

## 2. Related Work

### 2.1. Transfer Entropy

Transfer entropy (TE) is an information theoretic quantity that estimates the directed interaction, or information flow, between two dynamical systems (Zhu et al., 2015). It was introduced by Schreiber (2000) as a Wiener-causal measure within the framework of information theory. Therefore, TE is based on the assumption that a time series A causes a time series B if the information of the past of A, alongside the past of B, is better at predicting the future of B than the past of B alone. It is also based on the information theoretic concept of Shannon entropy:

(1) $$\begin{array}{l} H_{S}\left(X\right)=𝔼\left\{-\text{log}\left(p\left(x\right)\right)\right\}\approx -\underset{x}{\sum }p\left(x\right)\text{log}\left(p\left(x\right)\right), \end{array}$$

where *X* is a discrete random variable, *p*(·) is the probability mass function of *X*, and 𝔼{·} stands for the expected value operator. *H*_*S*_(*X*) quantifies the average reduction in uncertainty attained after measuring the values of *X*. By associating the improvement in prediction power of Wiener's definition of causality with the reduction of uncertainty measured by entropy, Schreiber arrived at the concept of TE (Vicente et al., 2011). Formally, TE measures the deviation from the following generalized Markov condition:

(2) $$\begin{array}{l} p\left(y_{t+1}|{\text{y}}_{t}^{m},{\text{x}}_{t}^{n}\right)=p\left(y_{t+1}|{\text{y}}_{t}^{m}\right), \end{array}$$

where ${\text{x}}_{t}^{n}\in {\mathbb{R}}^{n}$ and ${\text{y}}_{t}^{m}\in {\mathbb{R}}^{m}$ are Markov processes, of orders *n* and *m*, that approximate two time series $\text{x}={\left\{x_{t}\right\}}_{t=1}^{l}$ and $\text{y}={\left\{y_{t}\right\}}_{t=1}^{l}$, respectively, and *t* ∈ ℕ is a discrete time index. This deviation is quantified through the Kullback-Leibler divergence $\left(D_{KL}\left(p||q\right)=\underset{x}{\sum }p\left(x\right)\text{log}\left(p\left(x\right)/q\left(x\right)\right)\right)$ of the probability functions $p\left(y_{t+1}|{\text{y}}_{t}^{m},{\text{x}}_{t}^{n}\right)$ and $p\left(y_{t+1}|{\text{y}}_{t}^{m}\right)$:

(3) $$\begin{array}{l} TE\left(\text{x}\to \text{y}\right)=\underset{y_{t+1},{\text{y}}_{t}^{m},{\text{x}}_{t}^{n}}{\sum }p\left(y_{t+1},{\text{y}}_{t}^{m},{\text{x}}_{t}^{n}\right)\text{log}\left(\frac{p\left(y_{t+1}|{\text{y}}_{t}^{m},{\text{x}}_{t}^{n}\right)}{p\left(y_{t+1}|{\text{y}}_{t}^{m}\right)}\right). \end{array}$$

Therefore, TE measures whether the probability of a future value of **y** increases given the past values of **x** and **y**, as compared to the probability of that same future value of **y** given only the past of **y**.

In an attempt to better capture the underlying dynamics of the system that generates the observed data, i.e., the measured values of the random variables contained in the time series, TE is not usually defined directly on the raw data, but on its space state (Vicente et al., 2011). We can reconstruct such state space from the observations through time embedding. The most commonly used embedding procedure in the literature is Takens delay embedding (Takens, 1981). So that for a time series **x** its space state is approximated as:

(4) $$\begin{array}{l} {\text{x}}_{t}^{d}=\left(x\left(t\right),x\left(t-\tau \right),x\left(t-2\tau \right),\ldots ,x\left(t-\left(d-1\right)\tau \right)\right), \end{array}$$

where *d*, τ ∈ ℕ are the embedding dimension and delay, respectively. We can now express the TE in terms of the embedded data as:

(5) $$\begin{array}{l} TE\left(\text{x}\to \text{y}\right)=\underset{y_{t+1},{\text{y}}_{t}^{dy},{\text{x}}_{t}^{dx}}{\sum }p\left(y_{t+1},{\text{y}}_{t}^{dy},{\text{x}}_{t}^{dx}\right)\text{log}\left(\frac{p\left(y_{t+1}|{\text{y}}_{t}^{dy},{\text{x}}_{t}^{dx}\right)}{p\left(y_{t+1}|{\text{y}}_{t}^{dy}\right)}\right), \end{array}$$

where *dx, dy* ∈ ℕ. To generalize TE to interaction times other than 1, we rewrite Equation (5) as:

(6) $$\begin{array}{l} TE\left(\text{x}\to \text{y}\right)=\underset{y_{t},{\text{y}}_{t-1}^{dy},{\text{x}}_{t-u}^{dx}}{\sum }p\left(y_{t},{\text{y}}_{t-1}^{dy},{\text{x}}_{t-u}^{dx}\right)\text{log}\left(\frac{p\left(y_{t}|{\text{y}}_{t-1}^{dy},{\text{x}}_{t-u}^{dx}\right)}{p\left(y_{t}|{\text{y}}_{t-1}^{dy}\right)}\right), \end{array}$$

where *u* ∈ ℕ represents the interaction delay between the driving and the driven systems. The changes in the time indexing are necessary to guaranty that Wiener's definition of causality is respected (Wibral et al., 2013). Using the definition in Equation (1), we can also express Equation (6) as a sum of Shannon entropies:

(7) $$\begin{array}{l} TE\left(\text{x}\to \text{y}\right)=H_{S}\left({\text{y}}_{t-1}^{dy},{\text{x}}_{t-u}^{dx}\right)-H_{S}\left(y_{t},{\text{y}}_{t-1}^{dy},{\text{x}}_{t-u}^{dx}\right)\\ \;+H_{S}\left(y_{t},{\text{y}}_{t-1}^{dy}\right)-H_{S}\left({\text{y}}_{t-1}^{dy}\right). \end{array}$$

In practice, we must estimate the sum of Shannon entropies in Equation (7) from data. The most popular approach to do so, in neuroscience studies, is an adaptation for TE of the Kraskov-Stögbauer-Grassberger method for estimating mutual information (Kraskov et al., 2004; Dimitriadis et al., 2016). The method relies on a local approximation of the probability distributions needed to estimate the entropies from the distances of every data point to its neighbors, within a predefined neighborhood diameter. Also, it deals with the dimensionality differences in the data spaces in Equation (7) by fixing the number of neighbors in the highest dimensional space, the one spanned by $\left(y_{t},{\text{y}}_{t-1}^{dy},{\text{x}}_{t-u}^{dx}\right)$, and projecting the distances obtained there to the marginal (and lower dimensional) spaces so that they serve as neighborhood diameters in those. The Kraskov-Stögbauer-Grassberger estimator for TE is expressed as:

(8) $$\begin{array}{l} TE_{KSG}(\text{x}\to \text{y})=\psi (K)+E\{\psi \left(n_{{\text{y}}_{t-1}^{dy}}+1\right)\\ \;{-\psi \left(n_{y_{t}{\text{y}}_{t-1}^{dy}}+1\right)-\psi \left(n_{{\text{y}}_{t-1}^{dy}{\text{x}}_{t-u}^{dx}}\right)\}}_{t}, \end{array}$$

where ψ(·) stands for the digamma function, *K* ∈ ℕ is the selected number of neighbors in the highest dimensional space in Equation (7), 𝔼{·}_*t*_ represents averaging over different time points, and *n* ∈ ℕ is the number of points in the marginal spaces (Lindner et al., 2011).

An alternative approach for TE estimation relies on symbolic dynamics, a powerful tool for studying complex dynamical systems (Dimitriadis et al., 2012). The infinite number of values that can be attained by a given time series is replaced by a set of symbols through a symbolization scheme. We can then use the relative frequency of the symbols to estimate the joint and conditional probability distributions needed to compute TE (Dimitriadis et al., 2016). Given the space state reconstruction of a time series **x** (see Equation 4), we can arrange the elements in ${\text{x}}_{t}^{d}$ according to their amplitude, in ascending order, as follows:

(9) $$\begin{array}{l} x\left(t-r_{1}\tau \right)\leq x\left(t-r_{2}\tau \right)\leq \cdots \leq x\left(t-r_{d}\tau \right), \end{array}$$

where *r*_1_, *r*_2_, …*r*_*d*_ ∈ {0, 1, …, *d* − 1}, in order to obtain a symbolic sequence ${\text{s}}_{t}^{\text{x}}$:

(10) $$\begin{array}{l} {\text{x}}_{t}^{d}\to {\text{s}}_{t}^{\text{x}}\equiv \left(r_{1},r_{2},\ldots ,r_{d}\right), \end{array}$$

in what is known as ordinal pattern symbolization. Finally, we define the symbolic version of TE as:

(11) $$\begin{array}{l} TE_{Sym}\left(\text{x}\to \text{y}\right)=\underset{{\text{s}}_{t+1}^{\text{y}},{\text{s}}_{t}^{\text{y}},{\text{s}}_{t+1-u}^{\text{x}}}{\sum }p\left({\text{s}}_{t+1}^{\text{y}},{\text{s}}_{t}^{\text{y}},{\text{s}}_{t+1-u}^{\text{x}}\right)\text{log}\left(\frac{p\left({\text{s}}_{t+1}^{\text{y}}|{\text{s}}_{t}^{\text{y}},{\text{s}}_{t+1-u}^{\text{x}}\right)}{p\left({\text{s}}_{t+1}^{\text{y}}|{\text{s}}_{t}^{\text{y}}\right)}\right). \end{array}$$

We can rewrite Equation (11) in terms of Shannon entropies, as in Equation (7), and estimate the probability functions by counting the occurrences of the symbols (Dimitriadis et al., 2016).

The two methods described above rely on the use of plug-in estimators to approximate the probability distributions in the joint and marginal entropies involved in the definition of TE. Therefore, the so obtained TE depends on the quality of the estimated distributions and, consequently, on the performance of the plug-in estimator, be it based on a nearest neighbor distances approximation or a frequentist approach. Since the estimation of probability distributions can by itself be challenging, it would be desirable to be able to compute TE directly from the data, avoiding the intermediate stage of probability density estimation, as has been proposed for other information theoretic quantities (Giraldo et al., 2015).

### 2.2. Granger Causality

Granger Causality (GC), like TE, is a mathematical formalization of the concept of Wiener's causality, one that is widely used in neuroscience to asses effective connectivity (Seth et al., 2015). However, unlike TE, GC is not based on a probabilistic approach. The basic idea behind it is that for two stationary time series $\text{x}={\left\{x_{i}\right\}}_{i=1}^{n}$ and $\text{y}={\left\{y_{i}\right\}}_{i=1}^{n}$, if **x** causes **y**, then the linear autoregressive model:

(12) $$\begin{array}{l} y_{i}=\overset{o}{\underset{k=1}{\sum }}a_{k}y_{i-k}+e_{i}, \end{array}$$

where *o* ∈ ℕ is the model's order and *a*_*k*_ ∈ ℝ stands for the model's coefficients, will exhibit larger prediction errors *e*_*i*_ than a model that also includes past of observations of **x**; that is, a linear bivariate autoregressive model of the form:

(13) $$\begin{array}{l} y_{i}=\overset{o}{\underset{k=1}{\sum }}a_{k}^{\prime }y_{i-k}+\overset{o}{\underset{k=1}{\sum }}b_{k}x_{i-k}+e_{i}^{\prime }. \end{array}$$

where the coefficients *b*_*k*_ ∈ ℝ. The magnitude of the causal relation from **x** to **y** can then be quantified by the log ratio of the variances of the residuals or prediction errors (Seth, 2010):

(14) $$\begin{array}{l} GC\left(\text{x}\to \text{y}\right)=\text{log}\left(\frac{\text{var}\left(\text{e}\right)}{\text{var}\left({\text{e}}^{\prime }\right)}\right), \end{array}$$

where **e**,**e**′ ∈ ℝ^*n*−*o*^ are vectors holding the prediction errors, and var{·} stands for the variance operator. If the past of **x** does not improve the prediction of **y** then var(**e**) ≈ var(**e**′) and GC(**x** → **y**) → 0, if it does, then var(**e**) ≫ var(**e**′) and GC(**x** → **y**) ≫ 0. As defined above, GC is a linear bivariate parametric method that depends on the order *o* of the autoregressive model. Nonetheless, there are several variations of this basic formulation of GC that aim to capture nonlinear and multivariate relations in the data (Sameshima and Baccala, 2016). As a final remark, it is worth noting that although by definition TE has an advantage over GC by not assuming any a priori model for the interaction between the systems under study, the two are linked. As demonstrated in Barnett et al. (2009), they are entirely equivalent for Gaussian variables (up to a factor of 2). Because of this relationship and its widespread use we include a standard version of GC as a comparison method in our experiments.

## 3. Methods

### 3.1. Information Theoretic Learning From Kernel Matrices

Information-Theoretic-Learning (ITL) is a data-driven learning framework that employs information theoretic quantities as objective functions for supervised and unsupervised learning algorithms. However, instead of using the Shannon-based definition of entropy, ITL exploits the properties of a mathematical generalization of such a concept known as Renyi's α-order entropy. As explained before, Shannon entropy is defined as the expected value of the amount of information of the outcomes of a random variable. For a continuous random variable *X*, and using the linear averaging operator, we have that *H*(*X*) = 𝔼{*I*(*X*)} = ∫*p*(*x*)*I*(*x*)*dx*, where *I*(*x*) = −log(*p*(*x*)). Nonetheless, the linear mean is only a particular case of the average operator. In general, the expected value associated with a monotonic function *g*(*x*), with inverse *g*^−1^(*x*), is 𝔼{*x*} = *g*^−1^(∫*p*(*x*)*g*(*x*)*dx*). Furthermore, because of the postulate for additivity of independent events, in our case the possible choices for *g*(*x*) are restricted to only 2 classes: *g*(*x*) = *cx* and *g*(*x*) = *c*2^(1−α)*x*^. The former gives rise to the linear mean and therefore to the Shannon entropy, while the latter implies that:

(15) $$\begin{array}{l} H_{\alpha }\left(X\right)=\frac{1}{1-\alpha }\text{log}\left(\int p{\left(x\right)}^{\alpha }dx\right), \end{array}$$

with α ≠ 1 and α ≥ 0, which corresponds to Renyi's α entropy (Rényi, 1961; Principe, 2010). This parametric family of entropies encompasses the definition of Shannon entropy in the limiting case when α → 1. Furthermore, such generalization of Shannon's entropy allows emphasizing different characteristics of the data under analysis. In that sense, the parameter α can be tuned so that Renyi's entropy gives more weight to either mean behavior, by making α larger (α > 2), or to uncommon events, by making α smaller (α < 2) (Gao et al., 2011; Giraldo et al., 2015; Mammone et al., 2015).

In practice one must estimate entropy from discrete data. Given an *i.i.d*. sample of *n* realizations of a discrete random variable *X*, ${\left\{x_{i}\right\}}_{i=1}^{n}\subset {\mathbb{R}}^{d}$, the probability density function of *X* can be approximated through the Parzen density estimator as $\hat{p}\left(x\right)=\frac{1}{n}\overset{n}{\underset{i=1}{\sum }}\kappa \left(x,x_{i}\right)$, where κ(·, ·) ∈ ℝ stands for a positive definite kernel function. For the case of α = 2, and assuming a Gaussian kernel function, the Parzen approximation yields:

(16) $$\begin{array}{l} {\hat{H}}_{2}\left(X\right)=-\text{log}\left(\frac{1}{n^{2}}\overset{n}{\underset{i,j=1}{\sum }}\kappa \left(x_{i},x_{j}\right)\right), \end{array}$$

where the integral in Equation (15) has been replaced by a sum (Principe, 2010). The expression in Equation (16) can be rewritten in terms of a Gram matrix **K** ∈ ℝ^*n*×*n*^ as ${\hat{H}}_{2}\left(X\right)=-\text{log}\left(\frac{1}{n^{2}}\text{tr}\left(\text{K}\text{K}\right)\right)+C$, where **K** holds elements *k*_*ij*_ = κ(*x*_*i*_, *x*_*j*_), *C* ∈ ℝ^+^ accounts for the normalization factor of the Parzen window, and tr(·) stands for the matrix trace. From this result we can see that the Frobenius norm of the Gram matrix **K**, defined as ||**K**||^2^ = tr(**KK**), is related to an entropy estimator. In Giraldo et al. (2015) the authors generalize this notion. They extend it to other spectral norms, and introduce an entropy-like quantity with properties that closely resemble those of Renyi's entropy, while avoiding the estimation of probability distributions altogether. Given a Gram matrix **A** ∈ ℝ^*n*×*n*^ with elements *a*_*ij*_ = κ(*x*_*i*_, *x*_*j*_), a kernel-based formulation of Renyi's α-order entropy can be defined as:

(17) $$\begin{array}{l} H_{\alpha }\left(\text{A}\right)=\frac{1}{1-\alpha }\text{log}\left(\text{tr}\left({\text{A}}^{\alpha }\right)\right), \end{array}$$

where it holds that tr(**A**) = 1, and $0<H_{\alpha }\left(\text{A}\right)\leq H_{\alpha }\left(\frac{1}{n}\text{I}\right)$ with **I** the identity matrix. The power α of **A** can be obtained using the spectral theorem (Giraldo et al., 2015). Moreover, under this formulation, the joint entropy is defined as:

(18) $$\begin{array}{l} H_{\alpha }\left(\text{A},\text{B}\right)=H_{\alpha }\left(\frac{\text{A}\circ \text{B}}{\text{tr}\left(\text{A}\circ \text{B}\right)}\right)=\frac{1}{1-\alpha }\text{log}\left(\text{tr}\left({\left(\frac{\text{A}\circ \text{B}}{\text{tr}\left(\text{A}\circ \text{B}\right)}\right)}^{\alpha }\right)\right), \end{array}$$

where **B** ∈ ℝ^*n*×*n*^ is a Gram matrix holding the pairwise evaluation of the kernel function κ(·, ·) on an *i.i.d*. sample of *n* realizations of a second discrete random variable, and the operator ° stands for the Hadamard product. The joint entropy in Equation (18) can be extended to more arguments by computing the Hadamard product of all the corresponding kernel matrices. The above described kernel-based estimator of Renyi's entropy also satisfies the following set of conditions:

- $H_{\alpha }\left(\text{P}\text{A}{\text{P}}^{*}\right)=H_{\alpha }\left(\text{A}\right)$ for any orthonormal matrix **P** ∈ ℝ^*n*×*n*^.
- *H*_α_(*p***A**) is a continuous function for 0 < *p* ≤ 1.
- $H_{\alpha }\left(\frac{1}{n}\text{I}\right)=\text{lo}{\text{g}}_{2}n$, where **I** is the identity matrix.
- *H*_α_(**A**⊗**B**) = *H*_α_(**A**)+*H*_α_(**B**).
- If **AB** = **BA** = **0**; then for the function *g*(*x*) = 2^(α−1)*x*^, for α ≠ 1 and α ≥ 0, we have that $H_{\alpha }\left(t\text{A}+\left(1-t\right)\text{B}\right)=g^{-1}\left(tg\left(H_{\alpha }\left(\text{A}\right)\right)+\left(1-t\right)g\left(H_{\alpha }\left(\text{B}\right)\right)\right)$.

Besides, the functional in Equation (17) allows for the definition of conditional entropy and mutual information, provided the additional constraint that the kernels be infinitely divisible. Namely, the conditional entropy can be expressed as:

(19) $$\begin{array}{l} H_{\alpha }\left(\text{A}|\text{B}\right)=H_{\alpha }\left(\text{A},\text{B}\right)-H_{\alpha }\left(\text{B}\right), \end{array}$$

while the mutual information can be written as:

(20) $$\begin{array}{l} I_{\alpha }\left(\text{A};\text{B}\right)=H_{\alpha }\left(\text{A}\right)+H_{\alpha }\left(\text{B}\right)-H_{\alpha }\left(\text{A},\text{B}\right). \end{array}$$

### 3.2. Kernel-Based Renyi's Transfer Entropy

In this section, we introduce a novel TE estimator. We first generalize the concept of TE from Shannon entropies to Renyi's α-order entropies. Then, we propose a TE estimator using the entropy-like functionals derived in section 3.1, thus avoiding the intermediate step of probability distribution estimation in the computation of TE from discrete data. Given the state space reconstructions ${\text{x}}_{t}^{dx}$ and ${\text{y}}_{t}^{dy}$, of two time series **x** and **y**, the flow of information from **x** to **y**, for an interaction time *u*, corresponds to the deviation from the following equality: $p\left(y_{t}|{\text{y}}_{t-1}^{dy},{\text{x}}_{t-u}^{dx}\right)=p\left(y_{t}|{\text{y}}_{t-1}^{dy}\right)$. Now, instead of explicitly applying the definition of Kullback-Leibler divergence, as in the standard derivation of TE, we apply the expected value operator over the logarithm of the probability distributions, yielding:

(21) $$\begin{array}{l} {𝔼}_{y_{t},{\text{y}}_{t-1}^{dy},{\text{x}}_{t-u}^{dx}}\left\{-\text{log}\left(p\left(y_{t}|{\text{y}}_{t-1}^{dy},{\text{x}}_{t-u}^{dx}\right)\right)\right\}\\ \;={𝔼}_{y_{t},{\text{y}}_{t-1}^{dy}}\left\{-\text{log}\left(p\left(y_{t}|{\text{y}}_{t-1}^{dy}\right)\right)\right\}. \end{array}$$

Using the relations between conditional, joint and marginal probabilities, and rewriting the logarithms of the obtained quotients, we arrive at:

(22) $$\begin{array}{l} {𝔼}_{y_{t},{\text{y}}_{t-1}^{dy},{\text{x}}_{t-u}^{dx}}\left\{-\text{log}\left(p\left(y_{t},{\text{y}}_{t-1}^{dy},{\text{x}}_{t-u}^{dx}\right)\right)\right\}\\ \;-{𝔼}_{{\text{y}}_{t-1}^{dy},{\text{x}}_{t-u}^{dx}}\left\{-\text{log}\left(p\left({\text{y}}_{t-1}^{dy},{\text{x}}_{t-u}^{dx}\right)\right)\right\}\\ \;={𝔼}_{y_{t},{\text{y}}_{t-1}^{dy}}\left\{-\text{log}\left(p\left(y_{t},{\text{y}}_{t-1}^{dy}\right)\right)\right\}\\ \;-{𝔼}_{y_{t},{\text{y}}_{t-1}^{dy}}\left\{-\text{log}\left(p\left({\text{y}}_{t-1}^{dy}\right)\right)\right\}. \end{array}$$

The deviation from the above equality corresponds to transfer entropy, thus:

(23) $$\begin{array}{l} TE\left(\text{x}\to \text{y}\right)={𝔼}_{{\text{y}}_{t-1}^{dy},{\text{x}}_{t-u}^{dx}}\left\{-\text{log}\left(p\left({\text{y}}_{t-1}^{dy},{\text{x}}_{t-u}^{dx}\right)\right)\right\}\\ \;-{𝔼}_{y_{t},{\text{y}}_{t-1}^{dy},{\text{x}}_{t-u}^{dx}}\left\{-\text{log}\left(p\left(y_{t},{\text{y}}_{t-1}^{dy},{\text{x}}_{t-u}^{dx}\right)\right)\right\}\\ \;+{𝔼}_{y_{t},{\text{y}}_{t-1}^{dy}}\left\{-\text{log}\left(p\left(y_{t},{\text{y}}_{t-1}^{dy}\right)\right)\right\}\\ \;-{𝔼}_{y_{t},{\text{y}}_{t-1}^{dy}}\left\{-\text{log}\left(p\left({\text{y}}_{t-1}^{dy}\right)\right)\right\} \end{array}$$

From the general definition of entropy, *H*(*x*) = 𝔼{−log(*p*(*x*))}, and assuming an expected value associated with the function *g*(*x*) = *c*2^(1−α)*x*^, we can express TE as a sum of Renyi's α-order entropies:

(24) $$\begin{array}{l} TE_{\alpha }\left(\text{x}\to \text{y}\right)=H_{\alpha }\left({\text{y}}_{t-1}^{dy},{\text{x}}_{t-u}^{dx}\right)-H_{\alpha }\left(y_{t},{\text{y}}_{t-1}^{dy},{\text{x}}_{t-u}^{dx}\right)\\ \;+H_{\alpha }\left(y_{t},{\text{y}}_{t-1}^{dy}\right)-H_{\alpha }\left({\text{y}}_{t-1}^{dy}\right). \end{array}$$

In the limiting case when α → 1, Equations (7) and (24) are equivalent (TE_α_ yields the well-known TE). Finally, using the kernel-based formulation of Renyi's α-order entropy for marginal and joint probability distributions (Equations 17 and 18, respectively), we can estimate the TE_α_ from **x** to **y** as:

(25) $$\begin{array}{l} TE_{\kappa \alpha }\left(\text{x}\to \text{y}\right)=H_{\alpha }\left({\text{K}}_{{\text{y}}_{t-1}^{dy}},{\text{K}}_{{\text{x}}_{t-u}^{dx}}\right)-H_{\alpha }\left({\text{K}}_{y_{t}},{\text{K}}_{{\text{y}}_{t-1}^{dy}},{\text{K}}_{{\text{x}}_{t-u}^{dx}}\right)\\ \;+H_{\alpha }\left({\text{K}}_{y_{t}},{\text{K}}_{{\text{y}}_{t-1}^{dy}}\right)-H_{\alpha }\left({\text{K}}_{{\text{y}}_{t-1}^{dy}}\right), \end{array}$$

where the kernel matrices **K**_*y*_*t*__, ${\text{K}}_{{\text{y}}_{t-1}^{dy}}$, and ${\text{K}}_{{\text{x}}_{t-u}^{dx}}$ hold elements *k*_*ij*_ = κ(**a**_*i*_, **a**_*j*_), with *k*_*ij*_(·, ·) a positive definite, infinitely divisible kernel function. For matrix **K**_*y*_*t*__, *a*_*i*_, *a*_*j*_ ∈ ℝ are the values of the time series **y** at times *i* and *j*. In the case of matrix ${\text{K}}_{{\text{y}}_{t-1}^{dy}}$, the vectors ${\text{a}}_{i},{\text{a}}_{j}\in {\mathbb{R}}^{d}$ contain the space state reconstruction ${\text{y}}_{t}^{dy}$ of **y** at times *i* and *j*, adjusted according to the time indexing of TE. Likewise for ${\text{K}}_{{\text{x}}_{t-u}^{dx}}$.

## 4. Experiments

### 4.1. VAR Model

In order to test the ability of the TE_κα_ functional in Equation (25) to detect directed interactions under varying noise and data size conditions, we perform two experiments on simulated data. We generate synthetic data from a unidirectional bivariate autoregressive (AR) model of order 3:

(26) $$\begin{array}{l} {\text{z}}_{t}=\text{c}+\overset{3}{\underset{i=1}{\sum }}{\text{Q}}^{i}{\text{z}}_{t-i}+\varepsilon_{t}, \end{array}$$

where ${\text{z}}_{t}={\left(x_{t},y_{t}\right)}^{T}$ is a vector with the values of the simulated signals, **x** ∈ ℝ^*l*^ and **y** ∈ ℝ^*l*^, at time *t*, $\varepsilon_{t}\in {\mathbb{R}}^{2}$ is a vector of white noise values at time *t*, **c** ∈ ℝ^2^ is vector of constants, and

(27) $$\begin{array}{l} {\text{Q}}^{i}=\left(\begin{array}{c} q_{11}^{i}{\;q}_{12}^{i}\\ {\;q}_{21}^{i}{\;q}_{22}^{i} \end{array}\right);\;i=\left\{1,2,3\right\}, \end{array}$$

holds the model parameters. The directionality of the causal relation between the simulated time series is controlled by setting to 0 either the parameters $q_{12}^{i}$, to obtain a causal relation from **x** to **y**, or $q_{21}^{i}$ to obtain a causal relation in the opposite direction. The remaining parameters of the model are randomly selected. In order to assess the robustness of our method to different noise conditions, we add noise to the synthetic data as follows:

(28) $$\begin{array}{l} {\text{Z}}_{\eta }=\left(1-\gamma \right)\frac{\text{Z}}{||\text{Z}|{|}_{F}}+\gamma \frac{\Theta \Xi }{||\Theta \Xi |{|}_{F}}, \end{array}$$

where **Z** ∈ ℝ^2×*l*^ is a matrix containing the signals **x** and **y**, ||·||_*F*_ stands for the Frobenius norm, **Θ** ∈ ℝ^2×3^ is an instantaneous mixing matrix with random elements, and **Ξ** ∈ ℝ^3×*l*^ is a matrix containing 3 time series generated by 3 independent AR models of order 3 with otherwise random parameters, that represent multiple independent sources of noise and serve to simulate the effects of volume conduction. The parameter γ controls the relative strength of noise and signal (Dimitriadis et al., 2016). If γ is assigned a scalar value then signals **x** and **y** will exhibit symmetric noise, that is to say, they will have the same noise level. Alternatively, if γ is assigned a two-dimensional vector value, and the two elements of the vector are different, then the noise levels in **x** and **y** will be asymmetric (in this case, to be able to use Equation (28) we need to perform a column wise stacking of *l* copies of γ, and replace the scalar multiplication by a Hadamard product). In our first experiment we test both scenarios. First, we assign γ a scalar value that varies in the range from 0 to 1, in steps of 0.1, in order to simulate different symmetric levels of noise for signals of 512 data points. Then, to test the behavior of our TE estimator under asymmetric noise conditions, we assign γ a vector value and vary its two elements so as to form a two-dimensional grid, with each dimension ranging from 0 to 1, in steps of 0.1, for signals with the same number of data points as above. In the second experiment, we evaluate the impact of signal length on our method. To that end, we vary the length *l* of the noiseless simulated signals between 100 and 1,000 data points, in steps of 100 data points. For both experiments, that is to say, for each noise level (in the symmetric and asymmetric cases) and signal length, we estimate the accuracy for 10 realizations of 100 trials each. For each realization, the direction of interaction is chosen at random. The accuracy is defined in terms of a directionality index:

(29) $$\begin{array}{l} \Delta \lambda =\lambda \left(\text{x}\to \text{y}\right)-\lambda \left(\text{y}\to \text{x}\right), \end{array}$$

where λ(·) stands for any of the effective connectivity measures under consideration. Δλ indicates the preferred direction of information flow. It gets positive values for couplings from **x** to **y**, and negative values when **y** drives **x**. We use it to assess whether each effective connectivity measure correctly detects the chosen direction of interaction.

### 4.2. Modified Linear Kus Model

A method to estimate effective connectivity from multiple channel EEG data should be able to detect causal interactions among multiple signals coming from a connected network. With the aim of testing whether the proposed TE estimator could successfully reveal the presence, or absence, of such interactions in a known network, we use the modified version of the linear Kus model, introduced in Weber et al. (2017). It consists of 5 channels, connected through direct and indirect couplings (for a graphical representation of the model see Figure 4A). The input to the model is a time series containing real EEG data that is then contaminated with white Gaussian noise to obtain channel 1. Then, channel 1 is scaled and time-shifted by an interaction delay of 4 time units (δ = 4), and more white Gaussian noise is added, to generate channel 2. Channels 3 and 4 are generated in a similar fashion, while channel 5 consists only of white Gaussian noise. The following set of equations describes all the network interactions present in the model:

(30) $$\begin{array}{l} x_{1}\left(t\right)=\beta \left(t\right)+v\eta_{1}\left(t\right)\\ x_{2}\left(t\right)=0.4x_{1}\left(t-4\right)+v\eta_{2}\left(t\right)\\ x_{3}\left(t\right)=0.4x_{2}\left(t-4\right)+v\eta_{3}\left(t\right)\\ x_{4}\left(t\right)=0.4x_{2}\left(t-8\right)+v\eta_{4}\left(t\right)\\ x_{5}\left(t\right)=v\eta_{5}\left(t\right) \end{array}$$

where *x*_*j*_, β, and η_*j*_ stand for the 5 network channels, the input EEG data, and the added white Gaussian noise at time *t*, respectively. The parameter *v* is a scaling factor equivalent to a quarter of the variance of the time series. Additionally, external white Gaussian noise with zero mean and variance equal to *v* is added to all channels (Kus et al., 2004; Weber et al., 2017). It is worth noting that the indirect couplings in the model arise in two different ways. They can be the result of upstream dependences between the network's channels. For instance, channel 1 generates channel 2, which in turn generates channel 3, giving rise to an indirect coupling between channels 1 and 3. Indirect couplings can also arise from different time shifts applied to one channel in order to generate new channels. Such is the case of the indirect coupling between channels 3 and 4, which are generated by time-shifting channel 2 by 4 and 8 time units, respectively.

For our experimental set-up, we generate 1,000 trials of the modified Kus model, divided into 10 realizations. As input to the model, we use EEG data from the BCI Competition IV dataset 2a (for details about this dataset see section 4.3). Namely, we pool together the Fz channels from all subjects and trials in the dataset, and for each realization randomly select 100 of them (without repetition), to be used as inputs to the system. Then, we generate 100 trials of the modified Kus model, each consisting of a 5 channel network. Next, for all pair-wise combinations of channels in each trial, we estimate the directed interactions within the elements of the network using our method, and the other effective connectivity measures under consideration. Afterward, for each realization of 100 trials, we perform a permutation test, based on randomized trial surrogates, to determine which couplings or directed connections within the network are statistically significant at an alpha level of 5% (Lindner et al., 2011; Weber et al., 2017). The number of permutations in the test is set to 1,000. Finally, in order to asses the overall performance of each method, regarding the detection of the true connections in the modified Kus model, we compare the statistically significant connections per realization with the predefined connections in the network to obtain accuracy, sensitivity, and specificity values.

### 4.3. Motor Imagery

**Figure 1:**
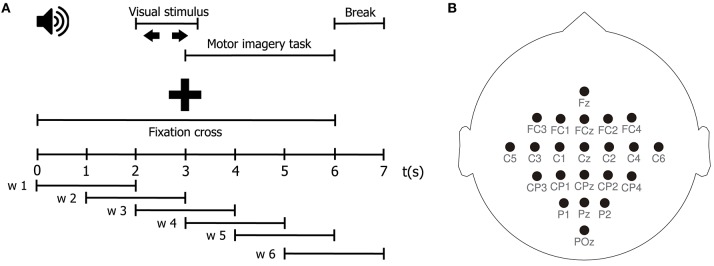
**(A)** Schematic representation of the MI protocol. **(B)** EEG channel montage used for the acquisition of the MI dataset.

Motor imagery (MI) is the process of imagining a motor action without any motor execution. During an MI task, a subject visualizes in his mind an instructed motor action, i.e., to move the right hand, without actually carrying it out. In order to test the performance of our TE estimator in the context of a BCI problem, we estimate effective connectivity features from EEG signals during two MI tasks. Our aim is twofold, first, to elucidate the directed interactions among EEG signals during the MI tasks; and second, to set up a classification system, based on such features, that allows discriminating between tasks. To those ends, we employ the publicly available BCI Competition IV database 2a^1^. This database consists of EEG data from 9 healthy subjects recorded while performing multiple trials of an MI protocol. Each trial starts with a fixed cross displayed on a computer screen, along with a beep. At second 2, an arrow pointing left, right, down or up (corresponding to the left hand, right hand, both feet, and tongue MI tasks) is presented as a visual cue on the screen for a period of 1.25 s. At second 3, the subjects perform the indicated MI task until second 6, when the cross vanishes from the screen. Then, the screen goes blank for 1 s indicating a short break. In this work, we use only 2 of the 4 MI tasks of the experimental paradigm, namely, left and right hand motor imagination. A schematic representation of the task is depicted in Figure 1A. The EEG signals are recorded from 22 Ag/AgCl electrodes positioned according to the international 10/20 placement system, as shown in Figure 1B, at a sampling rate of 250 Hz. Then, a 50 Hz Notch filter and a bandpass-filter between 0.5 and 100 Hz are applied to the recorded signals. The BCI Competition IV 2a database contains, for every subject, two separate sets of data obtained under the same experimental protocol: a Training dataset and a Testing dataset. The former is intended to be used to train the MI task classification system, while the latter should be used to test the performance of the trained system (Tangermann et al., 2012; Gómez et al., 2018).

For each subject, let $\text{Ψ}={\left\{{\text{X}}_{n}\in {\mathbb{R}}^{C\times M}\right\}}_{n=1}^{N}$ be the EEG set holding *N* trials of the MI tasks, with *C* = 22 channels, and *M* = 1,750 samples. Besides, let {1, 2}^*N*^ be a label set where the *n*-th element corresponds to the motor imagery task indicated for trial **X**_*n*_ (1 for right hand motor imagination, and 2 for left hand motor imagination). First, we perform a windowing procedure in order to both better capture the temporal dynamics of the MI task, which has several distinct stages, and to favor the stationarity of the EEG signals to be analyzed. We segment each EEG trial into six-time windows of 2 seconds with 50% overlapping, using a square window, obtaining six segments of equal length, as schematized in Figure 1A. The windowing procedure yields a set of matrices ${\left\{{\text{Z}}_{n}^{\text{w}}\in {\mathbb{R}}^{C\times L}\right\}}_{\text{w}=1}^{Q}$, where *Q* = 6, and *L* = 500. Our goal is thus to estimate the class label from effective connectivity features extracted from the segmented EEG trial ${\text{Z}}_{n}^{\text{w}}$. Afterward, we compute the surface Laplacian of each segmented trial using the spherical spline method for source current density estimation (Perrin et al., 1989). The surface Laplacian reduces the effects of volume conduction by attenuating low spatial frequency activity, and therefore, it also reduces the presence of spurious connections associated with it in connectivity analyses (Cohen, 2015; Rathee et al., 2017). Then, for each pairwise combination of channels ${\text{z}}_{c},{\text{z}}_{c^{\prime }}\in {\mathbb{R}}^{L}$, belonging to the spatially filtered version of ${\text{Z}}_{n}^{\text{w}}$, we estimate the effective connectivity $\lambda \left({\text{z}}_{c}\to {\text{z}}_{c^{\prime }}\right)$ to build a connectivity matrix **Λ** ∈ ℝ^*C*×*C*^. In the case when *c* = *c*′, we set $\lambda \left({\text{z}}_{c}\to {\text{z}}_{c^{\prime }}\right)=0$. Next, for time window w and for the *N* trials of the MI task, we obtain a set of connectivity matrices ${\left\{\Lambda_{n}^{\text{w}}\in {\mathbb{R}}^{C\times C}\right\}}_{n=1}^{N}$. After that, we apply vector concatenation to $\Lambda_{n}^{\text{w}}$ to yield a vector $\phi_{n}^{\text{w}}\in {\mathbb{R}}^{1\times \left(C\times C\right)}$. Then, we stack together the *N* vectors $\phi_{n}^{\text{w}}$, corresponding to each trial, to form a matrix **Φ**^w^ ∈ ℝ^*N*×(*C*×*C*)^. **Φ**^w^ holds all directed interactions, estimated through the effective connectivity measure λ, for time window w, for the entire EEG dataset Ψ.

After characterizing the EEG data, we set up our subject dependent MI task classification system. As mentioned before, the classification is carried out separately for each time interval or time window w. First, we perform two-sample Kolmogorov-Smirnov hypothesis tests over each of the *C* × *C* features of **Φ**^w^, after separating the data in function of their associated class labels. For each feature we obtain a *p*-value **ρ**, that we concatenate with those of all other features to generate a vector **ρ** ∈ (0, 1)^1×(*C*×*C*)^. Then, we use ρ to rank **Φ**^w^ according to the most discriminant features, that is, the directed interactions between pairs of channels with the smallest *p*-values. Next, we select the ranked features progressively and cumulatively, i.e., first only the most discriminant feature is selected, then the two most discriminant features, and so on. Afterward, the selected features are centralized, mapped to a new representation space trough PCA analysis, and input to a classification algorithm. After a performance evaluation, we choose *s* < *C*×*C* connectivity features to discriminate between the MI tasks of interest.

In order to evaluate the performance of the proposed classification system, we proceed in two different stages: a training-validation stage and a testing stage. For the training-validation stage, we first define a cross-validation scheme of 10 repetitions. For each repetition, 70% of the trials of the Training dataset are randomly assigned to a training set, and the remaining 30% to a validation set. Then, we characterize the training and validation sets and perform classification as described above, using a regularized linear discriminant analysis (LDA) classifier. All classification parameters are tuned at this stage, including the number of discriminant features *s*, and the percentages of retained variance of the PCA analyses. We adjust the parameters according to the classification accuracy, looking to improve the system's performance. Then, for the testing stage, we train an LDA classier using all trials from the Training dataset, and the parameters found during the training-validation stage. Next, we employ the trained classifier to predict the MI task class labels of the Testing dataset from effective connectivity features extracted from its EEG data. Finally, we compute accuracy values to quantify the performance of our classification system.

### 4.4. Parameter Selection for the Effective Connectivity Estimation Methods

We performed all the experiments mentioned above for two connectivity measures, namely TE and GC. For TE, we tested three different estimation strategies: the Kraskov-Stögbauer-Grassberger method (TE_KSG_), the symbolic version of TE based on ordinal pattern symbolization (TE_Sym_), and the proposed kernel-based Renyi's Transfer Entropy (TE_κα_). For the latter, we explored two values of the α parameter, α = {1.01, 2}, using as kernel function the radial basis function or RBF kernel (Liu et al., 2011):

(31) $$\begin{array}{l} \kappa \left({\text{a}}_{i},{\text{a}}_{j}\right)=\text{exp}\left(-\frac{||{\text{a}}_{i}-{\text{a}}_{j}|{|}^{2}}{2\sigma^{2}}\right). \end{array}$$

We used in-house Matlab implementations of the algorithms for GC, TE_Sym_, and TE_κα_^2^; while for TE_KSG_ we used the implementation provided by the open access toolbox TRENTOOL, a TE estimation and analysis toolbox for Matlab (Lindner et al., 2011).

Regarding the selection of parameters involved in the different effective connectivity estimation methods, we proceeded as follows: For the TE methods, the embedding delay τ was set to 1 autocorrelation time (ACT) (Vicente et al., 2011). The embedding dimension *d* and the interaction delay *u* were set in an experiment-dependent fashion, in most cases after a heuristic search intended to maximize performance. For all experiments, *d* was set to 3 after heuristic searches in the range *d* = {1, 2, …, 10}. For for the VAR model experiment and the MI tasks experiment *u* was set to 1, after heuristic searches in the ranges *u* = {1, 2, 3} and *u* = {1, 2, …, 100}, respectively. While for the Kus model experiment, *u* was set to 4, because that is the most common delay present in the model's network. The number of neighbors *K*, and the Theiler correction window in TRENTOOL's implementation of the TE_KSG_ algorithm were left at their default values of 4 and 1 ACT, respectively (Lindner et al., 2011). The bandwidth σ in the RBF kernel introduced in Equation (31), for the proposed TE_κα_ method, was set in each case as the median distance of the data (Schölkopf and Smola, 2002). The order of the autoregressive model *o* for GC was set to 3 for all experiments. In the case of the VAR model experiment *o* = 3 was chosen to coincide with the order of the data generation model, while for the Kus model and the MI tasks experiments it was the result of heuristic searches in the range *o* = {1, 3, 5, 7, 9}. Finally, the two values of the parameter α explored in all experiments were selected with the following rationale: as α → 1 Renyi's entropy tends to Shannon's entropy, so a value of α = 1.01 should allow for a better comparison with Shannon's entropy-based TE estimation strategies. Also, for Renyi's entropy a value of α = 2 is considered to be neutral to weighting (Giraldo et al., 2015), i.e., it does not emphasize or penalize rare events, which makes α = 2 a convenient choice when there is no previous knowledge about the values of the α parameter better suited for a particular application.

## 5. Results and Discussion

### 5.1. VAR Model

**Figure 2:**
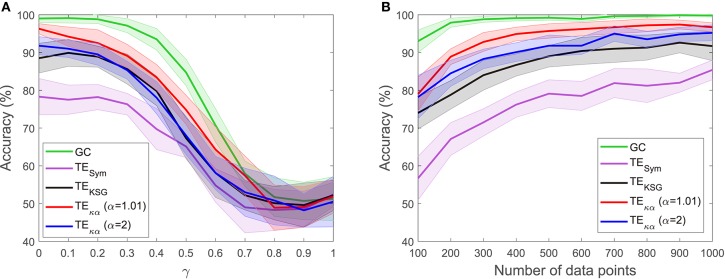
Accuracies in the detection of the preferred direction of information flow for synthetic data generated from a unidirectional bivariate autoregressive model of order 3: **(A)** for varying symmetric noise levels (as a function of the parameter γ), **(B)** for a varying number of data points.

**Figure 3:**
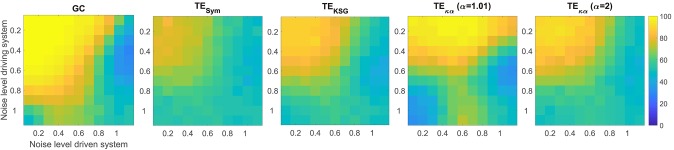
Average accuracies in the detection of the preferred direction of information flow for synthetic data, generated from a unidirectional bivariate autoregressive model of order 3 under asymmetric noise level conditions. The vertical axis displays the noise level for the driving time series, while the horizontal axis does so for the driven time series.

The experiments described in section 4.1 test whether the effective connectivity measures under consideration correctly detect the direction of interaction between two time series, under varying noise and data size conditions. Figures 2, 3 present the results of such experiments. Figure 2A shows the obtained average accuracies regarding the detection of the preferred direction of information flow as the scalar γ parameter in Equation (28), and thus the amount of symmetric noise added to the simulated signals, increases from 0 to 1. For all the methods tested the performance peaks for low noise levels and progressively falls as the noise level increases. At γ = 1 the average accuracies reach values of around 50%, which reflects the fact that for γ = 1 noise completely replaces the signals generated by the VAR model, and therefore no causal interaction is present. Figure 2B shows the average accuracies obtained with the effective connectivity measures tested as the number of data points of the VAR signals increases. In all cases, the performance is lowest for the lowest number of data points considered (100), and increases as the simulated signals become lengthier. This behavior is explained by the fact that a larger number of data points allows for a better estimation of the entropies (or their associated probability distributions) needed to compute TE, and for a better adjustment of the AR models in GC.

Figure 3 presents the average accuracies obtained with the effective connectivity measures studied under asymmetric noise conditions, in which the noise level varied independently for the driving and driven time series. This case is particularly interesting because asymmetries in the data, like different signal-to-noise ratios, different overall power or spectral details, and other asymmetries that can arise from volume conduction, have the potential to affect causality estimates (Haufe et al., 2013). In general, as the noise in any of the two time series increases, the accuracy in the detection of the preferred direction of information flow decreases. However, some of the methods tested produced spurious results when the noise levels differed, consistently estimating an incorrect interaction direction. This issue is not present in the results presented in Figure 2A for symmetric noise. Particularly, GC failed when the noise level was moderate for the driving time series and high for driven time series. Under those conditions GC estimates had an accuracy of around 30%, which means that for 70% of the simulated time series in that scenario GC estimated an incorrect direction of interaction. TE_κα_ for α = 1.01 also failed under the noise asymmetry conditions described above. Additionally, it failed when the noise level was high in the driving time series and low in the driven time series. On the flip side, it was more robust when the noise levels were reversed, that is, a low noise level in the driving time series and a high noise level in the driven time series. Our TE estimation method for α = 2 and the other approaches for TE estimation tested were not as affected by asymmetric noise.

For both VAR model experiments, GC outperforms TE, regardless of the TE estimation method. This result is not surprising, since the simulated data were generated using an AR model, and such models are at the core of the definition of GC. Furthermore, since the interactions present in the simulated data are purely linear a linear method, such as GC, is better suited to capture them than TE (Vicente et al., 2011). However, despite being outperformed by GC, within the proposed simulation framework, TE does reveal the direction of interaction of the data with high accuracy, albeit with marked estimation method dependent differences. Specifically, TE_κα_ exhibits the best performance of the TE estimation methods under study. In particular, for α = 1.01, it almost matches GC for the ideal conditions tested (a noiseless scenario, and a large number of data points). Interestingly, GC and TE_κα_, for α = 1.01, were the two methods most affected by asymmetric noise. Overall, within the tested simulation framework, our method fulfills two of the necessary conditions for a TE estimator apt for neuroscience applications (Vicente et al., 2011). Namely, it is robust to moderate levels of noise, represented in this case by a superposition of the signals of interest with those coming from unknown sources. This factor is at play in most noninvasive electrophysiological measurements such as EEG, which, to a large extent, contain unknown superpositions of many sources (Dimitriadis et al., 2016). Also, our estimator requires a smaller number of data samples to successfully determine the direction of interaction between a pair of signals, as compared with other TE estimators. The former is relevant because neuronal dynamics usually unfolds in periods of a few hundred milliseconds, which restricts the number of samples available to uncover any interaction of interest (Vicente et al., 2011). Additionally, the use of windowing to offset the effects of the non-stationarity of EEG signals further limits the number of data samples available to estimate TE (Cekic et al., 2018).

### 5.2. Modified Linear Kus Model

**Figure 4:**
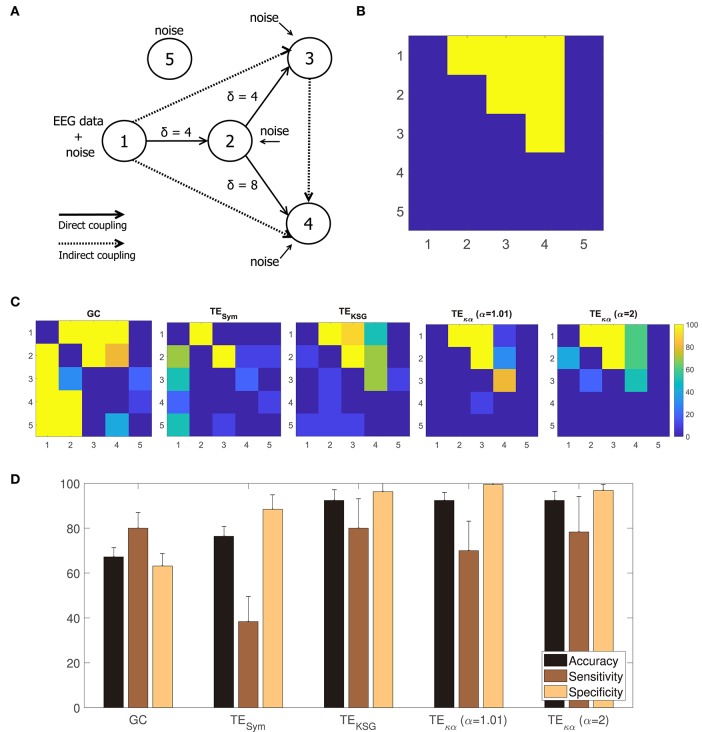
**(A)** Modified Kus model network coupling scheme. **(B)** Binary class matrix representation of the direct and indirect couplings in the modified Kus model network. **(C)** From left to right, statistically significant couplings according to a permutation test using trial randomized surrogates for GC, TE_Sym_, TE_KSG_, TE_κα_ (α = 1.01), and TE_κα_ (α = 2). **(D)** Accuracy, sensitivity, and specificity values obtained after comparing the statistically significant couplings shown in **(C)** with the matrix representation of the Kus model network presented in **(A)**.

Figure 4A shows a graphical representation of the 5 channel network constituting the modified linear Kus model. The solid and dashed lines represent the direct and indirect couplings present in the network, respectively; while the arrowheads indicate the direction of the causal relations introduced in the network by the time shifts δ. Figure 4B translates the network in Figure 4A to a binary class matrix representation. The positive class groups the direct and indirect connections among the network's channels. It is represented by the yellow elements, and their position, in the 5 × 5 connectivity matrix. On the other hand, the negative class is depicted in blue and represents non-existing interactions in the network. For instance, channel 1 drives channel 2; therefore element (1, 2) belongs to the positive class; but since the opposite is not true, element (2, 1) belongs to the negative class. Notice that all connections to and from channel 5 belong to the negative class. That is because channel 5 consists only of white Gaussian noise and is not coupled to the rest of the network.

In this work, the Kus model experiment is intended to evaluate if our method can detect causal interactions among multiple signals. Unlike the VAR-model experiment, in which we were solely interested in determining the correct direction of the model's causal interactions, this experiment also requires determining whether such interactions exist at all for any pair of signals within the model. To that end, we performed a permutation test, based on randomized surrogate trials, over the connectivity estimations, obtained with the methods studied, for each combination of channels (Lindner et al., 2011; Weber et al., 2017).

Figure 4C shows, from left to right, the percentage of statistically significant couplings in the 10 realizations of the experiment, according to the permutation test, for GC, TE_Sym_, TE_KSG_, TE_κα_ (α = 1), and TE_κα_ (α = 2). A visual inspection of Figure 4C reveals that the proposed TE_κα_ method and the TE_KSG_ method display the best performances. Namely, on average, for the 10 realizations of the experiment, the connectivity values estimated through those methods allow to better determine the actual connections present in the Kus model network. Therefore, their map of statistically significant couplings more closely resemble the actual Kus model connectivity matrix (Figure 4B). Note that TE estimators tested correctly detect both the presence and direction of the direct connections in the network for every realization, given that the time shift δ of the connection in question matches the chosen interaction delay *u*. That is the interactions from channel 1 to channel 2, and from channel 2 to channel 3, for which δ = 4, are successfully revealed. However, the direct connection between channels 2 and 4, for which δ = 8, proves more elusive. The TE_KSG_ method obtains statistically significant results for that specific coupling in 70% of the 10 realizations, while the TE_κα_ method does so for 60% of them. Interestingly, our method always detects the indirect connection from channel 1 to 3, despite an accumulated time shift of 8 time units. In addition, the proposed TE_κα_ method (α = 1.01) detects the indirect connection between channels 3 and 4 in more than 80% of the realizations. For the remaining connections, performance degrades for all the TE methods, probably as a result of both larger accumulated time shifts and the increasing amount of noise present in the network. It is also worth noting that our method does not point to the presence of directed interactions involving channel 5 for any realization.

Finally, by comparing the statistically significant couplings per realization with the binary class matrix representation of the Kus model network, we obtained accuracy, sensitivity, and specificity values for each of the effective connectivity estimation approaches tested. Figure 4D presents these results. The highest accuracies are achieved by the TE_κα_ and TE_KSG_ methods. Therefore, the proposed TE_κα_ method matches the performance of the TE_KSG_ algorithm regarding the detection of unknown causal interactions within a network from multi-channel data. Furthermore, the shown specificity values reflect the small number of false positives obtained with said methods. Along with the results observed in Figure 4C, this indicates that our approach seems to be suited to detect the couplings among the signals of a connected network with several interaction delays, while at the same time successfully identifying the pairs of non-interacting signals.

### 5.3. Motor Imagery

The MI tasks performed during the acquisition of the BCI IV database have a clear temporal structure, as depicted in Figure 1A. It follows that any characterization of the ensuing brain activity must reflect this structure. That is, since the visual cue indicating the MI task to be executed during a particular trial is presented to the subject at second 2, any information extracted from the EEG signals before that moment should not exhibit any discriminative power between tasks. Furthermore, since the subjects performed the MI task from seconds 3 to 6, this is the time period when the features extracted from the EEG signals of different tasks are expected to diverge. Since we aimed to test the ability of the proposed TE estimation method to elucidate the directed interactions among EEG signals during the MI tasks, and to determine whether those directed interactions allow discriminating between tasks, we can establish the compliance with the above-described temporal constraints as a necessary condition to achieve those aims.

**Figure 5:**
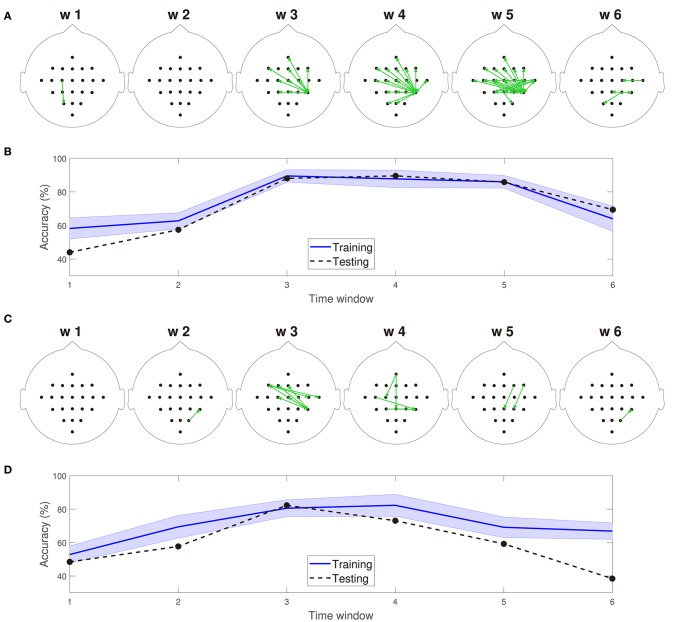
**(A)** Connections with statistically significant differences between the MI tasks for time windows 1–6 for subject 8. **(B)** Training and testing classification accuracies per time window for subject 8. **(C)** Connections with statistically significant differences between the MI tasks for time windows 1–6 for subject 9. **(D)** Training and testing classification accuracies per time window for subject 9. For visualization purposes, only 10% of the statistically significant connections, those with the smallest *p*-values, are depicted in **(A,C)**.

Figures 5A,C depict 10% of the directed connections estimated with the TE_κα_ method (α = 2), discriminated by time window, that present statistically significant differences between the left and right hand MI tasks for subjects 8 and 9, respectively. Such differences were assessed for each connection by applying a two-sample Kolmogorov-Smirnov hypothesis test to the connectivity data for the training dataset, after separating them in function of their associated class labels, and imposing a significance level of 0.01. We found few or no connections with statistically significant differences between conditions for time windows 1 and 2, which span from seconds 0 to 2, and 1 to 3, respectively. Then, for windows 3, 4, and 5 numerous connections to and from the centro-parietal area exhibit statistically significant task-dependent differences. Finally, the number of such connections decreases sharply for window 6, which covers seconds 5 to 7, and includes the break period after the MI task. Therefore, our method reveals directed interactions between EEG signals that present statistically significant differences between the right and left hand MI tasks, according to the temporal evolution of the MI protocol. Since the proposed classification system exploits the differences in the directed connections of each MI task to discriminate between them, its performance should also be conditioned by the same temporal constraints. Figures 5B,D display the training and testing classification accuracies, per time window for subjects 8 and 9, respectively. As expected, the classification system achieved its highest performances for the time windows during which the MI task was being executed by the subjects.

Tables 1, 2 present the highest accuracies achieved by the proposed classification system, for all subjects, and each of the effective connectivity methods studied. During the training-validation stage, the classifiers based on GC features and features extracted with TE_κα_, for α = 2, exhibited the highest average performances. However, during the testing stage the average performance of the GC-based classifier drops more than that of the TE_κα_-based classifier, which means that the latter generalizes better to new data. This points to a more stable identification of discriminant directed interactions across trials by our method as compared to other effective connectivity estimation approaches. Also, note that, in general, the TE_κα_-based classifier attains its best performances for the time windows corresponding to the execution of the MI task. Here, we must highlight the fact that the accuracies presented in Tables 1, 2 fall short of those obtained with feature extraction strategies other than effective connectivity analyses, such as common spatial patterns (Elasuty and Eldawlatly, 2015; Gómez et al., 2018; Li et al., 2018). This underperformance of connectivity-based analysis for MI tasks discrimination has been linked to the difficulties of measuring local or short-range connectivities, such as those expected to appear among different zones of the motor areas during MI tasks, due to volume conduction effects (Rathee et al., 2017). Interestingly, the results obtained with the classifiers based on features extracted with our method, and with the other effective connectivity measures studied, tend to coincide with those of classifiers based on alternative characterization strategies, in terms of the ranking of the performances per subject; that is, subjects 8, 9, or 3 present the highest performances, while subjects 2, 5, or 6 exhibit the lowest ones (Elasuty and Eldawlatly, 2015; Liang et al., 2016; Gómez et al., 2018; Li et al., 2018).

**Table 1:** Average training accuracy [%] for the window (**w**) with the best performance.

**Subject**	**TE_********κα********_** (**α** = **2**)		**TE_********κα********_** (**α** = **1.01**)		**TE_*******KSG*******_**		**TE_*******Sym*******_**		**GC (order 3)**	
	**acc**	**(w)**	**acc**	**(w)**	**acc**	**(w)**	**acc**	**(w)**	**acc**	**(w)**
s 01	71.2 ± 6.4	(3)	**76.9** ± **6.7**	(3)	61.2 ± 7.3	(2)	61.0 ± 7.9	(4)	73.8 ± 7.1	(3)
s 02	56.4 ± 4.9	(2)	58.1 ± 7.1	(1)	58.6 ± 7.5	(2)	59.2 ± 8.6	(3)	**65.7** ± **8.3**	(6)
s 03	81.2 ± 3.5	(4)	77.9 ± 6.3	(4)	75.7 ± 6.8	(4)	83.6 ± 3.3	(4)	**83.8** ± **7.2**	(4)
s 04	**63.8** ± **4.3**	(2)	60.0 ± 7.0	(3)	63.5 ± 6.5	(1)	62.3 ± 6.7	(5)	60.0 ± 3.9	(4)
s 05	**69.7** ± **3.8**	(3)	64.9 ± 6.4	(4)	67.9 ± 8.8	(3)	60.0 ± 6.4	(4)	67.2 ± 7.5	(4)
s 06	**65.4** ± **5.6**	(3)	62.9 ± 7.6	(4)	62.6 ± 11.6	(4)	58.6 ± 7.6	(2)	65.4 ± 6.7	(3)
s 07	70.0 ± 8.3	(3)	**73.7** ± **4.1**	(3)	65.6 ± 6.9	(3)	64.4 ± 6.8	(5)	72.4 ± 8.0	(3)
s 08	**89.5** ± **3.7**	(3)	80.5 ± 4.4	(4)	66.0 ± 5.2	(5)	78.5 ± 5.9	(4)	87.8 ± 3.8	(4)
s 09	82.3 ± 6.6	(4)	73.4 ± 7.7	(3)	70.6 ± 6.2	(4)	**82.6** ± **4.6**	(3)	75.7 ± 5.8	(4)
AVG	72.2 ± 5.2		69.8 ± 6.4		65.7 ± 7.4		67.8 ± 6.4		**72.4** ± **6.5**	

*The bold values indicate the highest accuracies obtained for each subject*.

**Table 2:** Testing accuracy [%] for the window (**w**) with the best performance.

**Subject**	**TE_********κα********_** (**α** = **2**)		**TE_********κα********_** (**α** = **1.01**)		**TE_*******KSG*******_**		**TE_*******Sym*******_**		**GC (order 3)**	
	**acc**	**(w)**	**acc**	**(w)**	**acc**	**(w)**	**acc**	**(w)**	**acc**	**(w)**
s 01	**70.9**	(3)	68.1	(3)	58.9	(5)	61.0	(2)	67.4	(4)
s 02	54.2	(6)	57.7	(6)	**59.9**	(1)	56.3	(6)	58.5	(6)
s 03	80.3	(4)	73.0	(4)	67.2	(3)	**81.0**	(4)	70.8	(4)
s 04	**63.8**	(3)	61.2	(4)	53.4	(5)	57.8	(5)	57.8	(3)
s 05	53.3	(3)	**53.3**	(4)	51.9	(2)	53.3	(3)	51.9	(6)
s 06	59.3	(3)	**62.0**	(4)	60.2	(2)	54.6	(3)	53.7	(2)
s 07	**65.7**	(3)	62.1	(3)	58.6	(6)	60.0	(1)	59.3	(6)
s 08	**89.6**	(4)	73.1	(3)	64.2	(4)	82.8	(4)	79.9	(4)
s 09	**82.3**	(3)	76.9	(3)	62.3	(4)	73.8	(4)	70.8	(4)
AVG	**68.8** ± **12.9**		65.3 ± 7.9		59.6 ± 4.8		64.5 ± 11.5		63.3 ± 9.3	

*The bold values indicate the highest accuracies obtained for each subject*.

**Figure 6:**
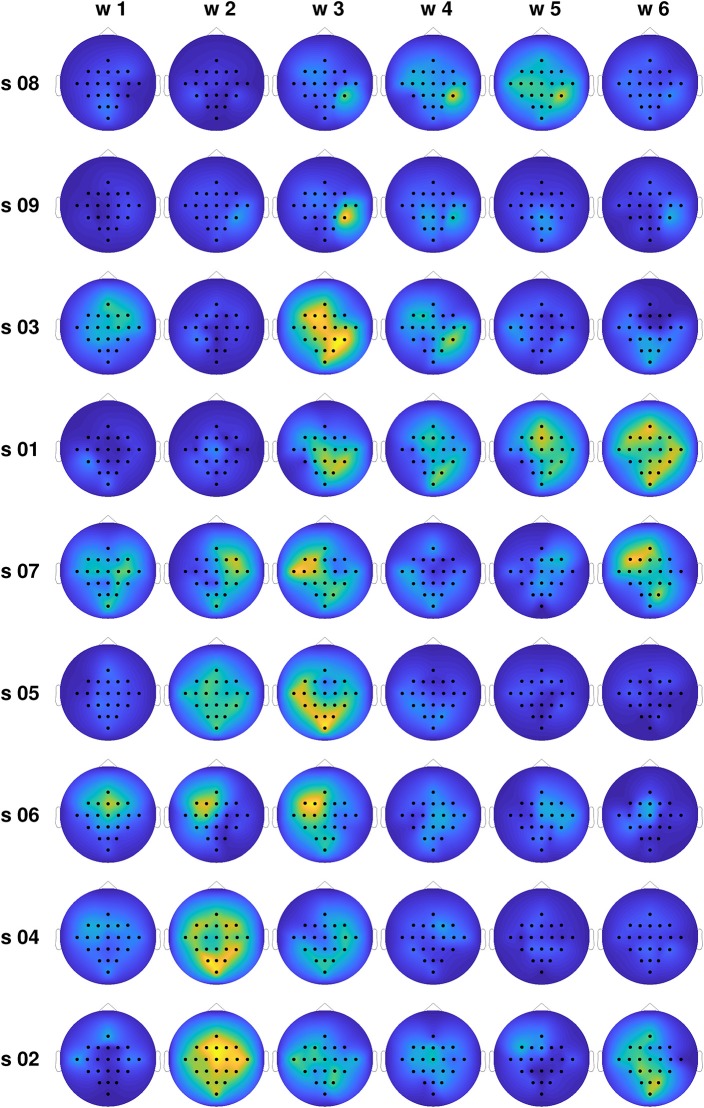
Normalized average differences in the total information flow coming into each channel for the training set, for all subjects and time windows. Large differences are coded in yellow, while small differences are presented in blue.

In order to gain insight into the large differences in classification performance observed for the different subjects, we computed the average differences in the total information flow coming into each channel, estimated through the proposed TE_κα_ method (α = 2), for all subjects and time windows. Namely, for each trial, we obtained the total information flow coming into a particular channel as the sum of all directed interactions targeting that channel, then averaged that magnitude across all trials of the same MI task, and finally subtracted the averages of the left and right MI tasks. Figure 6 shows the obtained results. The subjects are organized in descending order according to the classification accuracies presented in Table 1. For the subjects at the top of the plot, we observed a clear temporal evolution, with small variations between the information flow of both tasks for time windows 1 and 2, and large localized differences during the time windows corresponding to MI execution. We can also observe a trend regarding the spatial location of the information flow differences. For the top 4 subjects, particularly for time window 3, they are centered around the centro-parietal region, specifically channel CP4. For the subjects at the bottom of the plot, the same temporal and spatial patterns are not present. Here, it is worth noting that we have focused our analyses on the differences in the obtained effective connectivities for the left and right MI tasks, instead of analyzing the connectivities that arise for each task as compared with the resting state (Gong et al., 2018). Bearing this in mind, and considering the physiological interpretation of MI which states that motor imagination mainly activates motor representations in the premotor cortex and the parietal area (Hétu et al., 2013), we can argue that it is the differences in the information flow to and from the right parietal cortex, during the activation associated with MI, which allowed us to discriminate between tasks for a subset of the subjects.

The above results, and those of sections 5.1 and 5.2, show that the proposed TE_κα_ method is apt for TE estimation from neuroscience data. Regarding the requirements outlined in section 1, we have shown that our TE estimator is robust to moderate levels of noise and performs satisfactorily under data size constrains. The third requirement, concerning the reliability of the estimator when dealing with high-dimensional spaces, is readily taken care of by the intrinsic capacity of kernels to deal with such spaces (Schölkopf and Smola, 2002). Nonetheless, our approach also has shortcomings, which we will discuss in the following.

First, we must note that the exponentiation operation in Equation (17), central to the kernel-based approximation of Renyi's entropy, makes our TE estimator ill-suited for the analysis of long time series (i.e., time series with several thousands of data points) due to the increase in computational cost. This is especially true for non-integer values of α. Furthermore, our approach also exhibits limitations inherent to the concept of TE (Vicente et al., 2011). Namely, the definition of causality underlying TE is observational, so unobserved common causes cannot be analyzed. This shortcoming encompasses the different delay driving problem. Given three variables, this problem occurs when the first variable drives the two remaining variables but each with a different delay, giving rise to an indirect casualty relation between the second and the third variables that cannot be identified as spurious in bivariate connectivity analyses (Cekic et al., 2018). Systems related by a deterministic map, such as those that are completely synchronized, cannot be analyzed either. Additionally, the fact that TE is model-free implies that while TE provides information about the directed or causal interactions among data, it does not give any further insight into the nature of those interactions. Furthermore, TE assumes at most weak non-stationarities in the data, so strong non-stationarities pose a challenge for its estimation; although progress has been made in that regard (Wollstadt et al., 2014). Finally, by using Renyi's entropy measures of order α to define TE, instead of Shanon's entropy, we gain flexibility regarding the characteristics of the data we wish to highlight, by having at our disposal an entire parametric family of entropies. As observed in our results, the choice of the parameter α indeed influences the performance of the TE_κα_ estimator. It becomes more or less successful at uncovering the interactions of interest as a function of α. The flip side of this flexibility is that in practice α becomes one more parameter to select. In general, the choice of α should be associated with the task goal (Principe, 2010). For Renyi's entropy a large α emphasizes slowly changing features (Giraldo et al., 2015). Particularly, α > 2 characterizes mean behavior, while α < 2 emphasizes rare events or multiple modalities, and α = 2 is neutral to weighting.

## 6. Conclusion

In this work, we proposed a new TE estimator based on Renyi's entropy of order α, which we approximate through positive definite kernel matrices. Our data-driven method, termed TE_κα_, sidesteps the probability distribution estimation stage involved in the computation of TE from discrete data, thus avoiding the challenges associated with it. We tested the performance of our method on two different synthetic datasets, and on an EEG-database obtained under an MI paradigm. We compared it with that of state-of-the-art methods for TE estimation, as well as with that of GC, another commonly used brain effective connectivity measure. Our results show that the proposed TE estimator successfully detects the presence and direction of Wiener-causal interactions between a pair of signals, exhibiting robustness to varying noise levels and number of available data samples, and to the presence of multiple interaction delays within a connected network. Furthermore, our method revealed discriminant spatiotemporal patterns for the MI tasks, that are consistent across the top performing subjects, and which follow the temporal constraints imposed by the MI experimental paradigm. For all the performance evaluation metrics employed, the proposed kernel-based TE estimation method is competitive with the state-of-the-art. As future work, we will look into developing a data-driven approach to select α, as well as the kernel bandwidth in the RBF function. Also, we will work toward obtaining a spectral representation for TE using the proposed kernel-based estimator.

## Data Availability Statement

The datasets generated for this study are available on request to the corresponding author.

## Author Contributions

ID: theoretical development and coding of the proposed kernel-based TE estimation method, synthetic data and motor imagery database tests and analyses, and manuscript writing. AA-M: theoretical development of the proposed method, and manuscript writing support. AO-G: design of the tests and analyses carried out, and manuscript writing support.

### Conflict of Interest

The authors declare that the research was conducted in the absence of any commercial or financial relationships that could be construed as a potential conflict of interest.

## Footnotes

^1^http://www.bbci.de/competition/iv/desc_2a.pdf

^2^Available at: https://github.com/ide2704/Kernel_Renyi_Transfer_Entropy
